# Immune triggers preceding neuralgic amyotrophy

**DOI:** 10.1111/ene.16462

**Published:** 2024-10-04

**Authors:** Davide Sparasci, Lenka Schilg‐Hafer, Bettina Schreiner, Olivier Scheidegger, Anne‐Kathrin Peyer, Agustina Maria Lascano, Alex Vicino, Bernhard Friedrich Décard, Pinelopi Tsouni, Andrea Monika Humm, Enea Pianezzi, Giulia Zezza, Thomas Hundsberger, Anelia Dietmann, Hans H. Jung, Thierry Kuntzer, Einar Wilder‐Smith, Gladys Martinetti‐Lucchini, Orlando Petrini, Stefano Fontana, Peter Gowland, Christoph Niederhauser, Claudio Gobbi, Paolo Ripellino

**Affiliations:** ^1^ Department of Neurology Neurocenter of Southern Switzerland EOC Lugano Switzerland; ^2^ Department of Neurology Cantonal Hospital St Gallen Switzerland; ^3^ Department of Neurology University and Hospital Zurich Zurich Switzerland; ^4^ Department of Neurology, Inselspital Bern University Hospital and University of Bern Bern Switzerland; ^5^ Department of Neurology University Hospital and University of Basel Basel Switzerland; ^6^ Neurology Division, Department of Clinical Neuroscience, University Hospitals of Geneva and Faculty of Medicine University of Geneva Geneva Switzerland; ^7^ Nerve‐Muscle Unit, Neurology Service, Department of Clinical Neurosciences Lausanne University Hospital and University of Lausanne Lausanne Switzerland; ^8^ Cantonal Hospital Lucerne Switzerland; ^9^ Department of Neurology Hôpital du Valais Sion Switzerland; ^10^ Neurology Unit, Department of Medicine HFR Fribourg Cantonal Hospital Fribourg Switzerland; ^11^ Laboratory of Microbiology EOC Bellinzona Switzerland; ^12^ University of Applied Sciences and Arts of Southern Switzerland Bellinzona Switzerland; ^13^ Blood Transfusion Service SRC Southern Switzerland Lugano Switzerland; ^14^ Interregional Blood Transfusion SRC Bern Switzerland; ^15^ Institute for Infectious Diseases University of Bern Bern Switzerland; ^16^ Faculty of Biomedical Sciences Università della Svizzera Italiana Lugano Switzerland

**Keywords:** immune trigger, infection, neuralgic amyotrophy, Parsonage–Turner syndrome, vaccination

## Abstract

**Background and purpose:**

Infections and vaccinations have been identified as potential immunological triggers of neuralgic amyotrophy (NA), but the exact type and frequency of the preceding agents is unknown.

**Methods:**

This was a multicentre, prospective, observational, matched case–control study. NA was diagnosed by neuromuscular experts according to validated clinical criteria and electrodiagnostic studies. Clinical data and biological samples of NA patients were collected within 90 days from disease onset between June 2018 and December 2023. All NA patients were asked about prior infection and vaccination in the month before disease onset. Serological tests for hepatitis E virus, human immunodeficiency virus, severe acute respiratory syndrome coronavirus 2, Epstein–Barr virus, cytomegalovirus, parvovirus B19, varicella‐zoster virus, *Borrelia burgdorferi*, *Mycoplasma pneumoniae* and *Bartonella henselae* were performed in a central laboratory. Each case was matched with a healthy control for age, sex, place of residence and time of blood collection.

**Results:**

Fifty‐seven patients and corresponding controls were included. The mean age was 45 years for both groups. NA onset was preceded by a symptomatic infectious trigger confirmed by microbiological tests in 15/57 (26.3%) patients. Coronavirus disease 2019 vaccination was considered a potential trigger in 7/57 (12.3%) subjects. An acute viral infection was associated with a bilateral involvement of the brachial plexus (*p* = 0.003, Cramèr's *V* = 0.43).

**Conclusions:**

Confirmed immune triggers (infection or vaccination) preceded disease onset in 22/57 (38.6%) NA cases. We suggest to test NA patients in the acute phase for intracellular antigens, especially in the case of concomitant bilateral involvement and hepatitis.

## INTRODUCTION

Neuralgic amyotrophy (NA), also known as Parsonage–Turner syndrome, is an acute and monophasic brachial plexus disorder characterized by severe shoulder pain, followed by muscle weakness and atrophy of the affected limb [1]. It is considered a rare disease, but more detailed observations in the Netherlands estimated an annual incidence of 1:1000 [2], young to middle‐aged male adults being mostly affected [3]. The pathophysiology of NA is probably multifactorial, combining genetic, immune and mechanical (e.g., exercise) factors [3]. Preceding events possibly related to NA include infections, vaccinations, strenuous exercise, pregnancy and surgery [1, 3, 4, 5].

All studies published so far, however, lacked appropriate controls; in addition, serological blood tests to identify the pathogen were not routinely performed, because most NA cases were diagnosed only several months after symptom onset [6] and not during the acute phase. Therefore, it is still unclear which of the most frequent pathogens may be associated with NA, and consequently which serological tests should be carried out in the acute setting.

This study aimed to determine the prevalence of vaccinations and the most common infectious agents preceding NA in a Swiss cohort.

## METHODS

This was a prospective, multicentre, observational, matched case–control study.

### Participants

Patients older than 18 years with NA were enrolled within 90 days from disease onset from June 2018 to December 2023 in 11 Swiss neuromuscular centres. In addition to clinical criteria (subacute onset within hours; initial pain with visual analogue scale score >7/10; multifocal distribution of neurological injury centred on the brachial plexus; monophasic course with slow recovery) [2], each NA diagnosis was confirmed by electrodiagnostic studies. When symptoms/signs were bilateral but asymmetric, the patient was considered as having a bilateral NA.

Blood samples of all patients were subjected to serology and polymerase chain reaction (PCR) diagnostics, as appropriate, for selected viral or bacterial pathogens. In addition, all patients were asked about symptoms of possible recent infection (fever, diarrhoea, flu‐like syndrome, angina, cough and respiratory symptoms, jaundice, nausea, vomit) and their vaccination status. Any vaccination received within 2 weeks before the onset of neurological symptoms was considered to be potentially related to the disease.

Healthy blood donors were chosen as controls and matched on sex, age (±5 years), canton of residence in Switzerland and time of blood collection (±6 months from the collection date).

All NA cases underwent a 3‐month follow‐up visit.

### Laboratory testing

Serum samples from NA cases were collected before any treatment within 90 days after the onset of NA symptoms during routine diagnostic procedures and stored at −80°C for serological and PCR diagnostics. All sera from patients and controls were tested at the Microbiology Laboratory of the Canton Hospital of southern Switzerland for immunoglobulin (Ig) M and IgG antibodies of hepatitis E virus (HEV), human immunodeficiency virus (HIV), severe acute respiratory syndrome coronavirus 2 (SARS‐CoV‐2), Epstein–Barr virus (EBV), cytomegalovirus (CMV), parvovirus B19, varicella‐zoster virus (VZV), *Borrelia burgdorferi*, *Mycoplasma pneumoniae* and *Bartonella henselae*.

For HEV, the enzyme‐linked immunosorbent assay (ELISA) Wantai HEV‐IgG and Wantai HEV‐IgM kits (Eurobio, Les Ulis, France) were used according to the manufacturer's instructions. ELISA results are ratios of the sample optical density (OD) divided by the cut‐off OD provided by the manufacturer. For both IgM and IgG antibodies OD ratios >1.1 indicated a positive result and ratios <0.9 a negative result. Borderline OD ratios (0.9–1.1) were classified as positive if the diagnosis was supported by positive anti‐HEV IgG antibodies. HEV real time PCR (RT‐PCR) was performed in serum samples of cases and controls with anti‐HEV IgM+ and/or increased liver enzymes, using the RealStar kit (Altona Diagnostics, Hamburg, Germany), according to the manufacturer's instructions.

The CMV and EBV assays were carried out using the chemiluminescent microparticle capture immunoassay on an Alinity analyser (Abbott Diagnostic Inc., Abbott Park, IL, USA). A recent CMV infection was defined as IgM positivity with negative IgG or IgG with low avidity. CMV IgG avidity was tested only if CMV IgM was positive. A recent primary EBV infection was defined as a positive viral capsid antigen IgM, alone or with viral capsid antigen IgG positivity, and no antibody to EBV nuclear antigen. HIV infection was diagnosed using the chemiluminescent microparticle capture immunoassay for the qualitative determination of antibodies and the p24 antigen of HIV type 1 and type 2 (fourth generation test).

The quantitative test for IgG antibodies against the SARS‐CoV‐2 spike protein was performed on an Alinity analyser according to the manufacturer's instructions. A recent SARS‐CoV‐2 infection was established only in the case of positive RT‐PCR on nasopharyngeal swab test, using the Cobas 6800 SARS‐CoV‐2 test (Roche Molecular Systems, Rotkreuz, Switzerland).

IgM and IgG antibodies against *Borrelia burgdorferi*, *Mycoplasma pneumoniae*, parvovirus B19 and VZV were determined on a LIAISON XL (DiaSorin, Saluggia, Italy) using chemiluminescent immunoassay technology immunoassays [7], and IgM antibodies against VZV using a specific immunofluorescent assay (Euroimmun, Abbott Park, IL, USA). IgM and IgG antibodies against *Bartonella henselae* were determined by immunofluorescent assays (Fuller Laboratories, Fullerton, CA, USA). Cases and controls resulting positive for IgM for borreliosis underwent a confirmatory test with western blot analysis.

The liver enzymes alanine aminotransferase (ALT) and gamma‐glutamyltransferase (GGT) were tested in cases at the same time as the serological tests, that is, in each case within 90 days of the onset of symptoms. Values of ALT above 50 U/L and of GGT above 71 U/L were considered pathological.

### Statistical analysis

Prior to analysis, data were checked for goodness of matching between cases and their matched controls.

Continuous data (age, liver enzymes) are presented descriptively as mean, standard deviation (SD), minimum and maximum. Frequency data are shown as counts and percentages, and pairwise associations between variables were computed using contingency tables. Fisher's exact test was used to detect associations, with Cramèr's *V* as a measure of association. Odds ratios (ORs) and the corresponding 95% confidence intervals were computed with MacNemar's test. Stata Version 18 (StataCorp LCC, College Station, TX, USA) was used for all statistical analyses and data presentation.

## RESULTS

### Demographics features

Fifty‐seven NA patients were enrolled and matched with corresponding controls chosen amongst blood donors. Study population characteristics of NA cases and controls were comparable (Table 1).

**TABLE 1:** Demographics of the study population.

	Cases (*N* = 57)	Controls (*N* = 57)	Total (*N* = 114)
Females			
*n* (%)	25 (43.86)	25 (43.86)	50 (43.86)
Age [years]	43.84 (15.34) [18–72]	43.80 (14.46) [20–70]	43.82 (14.76) [18–72]
Males			
*n* (%)	32 (56.14)	32 (56.14)	64 (56.14)
Age [years]	48.91 (16.72) [18–80]	48.88 (16.62) [21–80]	48.89 (16.54) [18–80]

*Note*: Age is presented as mean (standard deviation) [minimum–maximum], gender as *n* (percent). Differences between cases and controls are not statistically significant for age or gender proportion.

### Serology

The onset of NA was preceded by an anamnestic (medical history) immune trigger in 33/57 (57.9%) patients. An acute laboratory proven infection as a potential trigger was detected in 15/57 cases (26.3%), whilst a coronavirus disease 2019 (COVID‐19) vaccination in the 2 weeks preceding symptom onset was reported in 7/57 (12.3%) patients (Table 2). Moderna COVID‐19 vaccine (mRNA‐1273) had been used to vaccinate three and Pfizer/BioNTech COVID‐19 vaccine (BNT162b2) four patients. There was no laboratory evidence of recent infections in matched controls.

**TABLE 2:** Types of immune trigger, *n* (%).

	Cases	Controls
Viral infections		
HEV	8 (14.0)	–
SARS‐CoV‐2	1 (1.7)	–
EBV	2 (3.5)	–
Parvovirus B19	1 (1.7)	–
VZV	1 (1.7)	–
Bacterial infections		
*Mycoplasma pneumoniae*	2 (3.5)	–
Vaccination		
SARS‐CoV‐2 vaccine	7 (12.3)	–

Abbreviations: EBV, Epstein–Barr virus; HEV, hepatitis E virus; SARS‐CoV‐2, severe acute respiratory syndrome coronavirus 2; VZV, varicella zoster virus.

**FIGURE 1:**
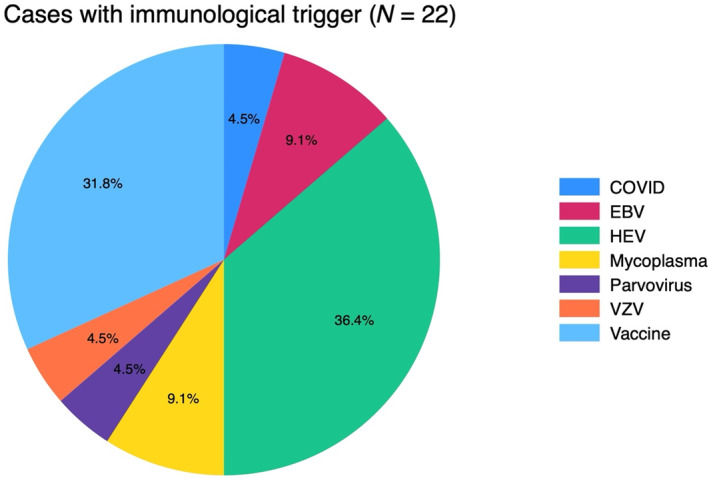
Cases with identified immunological trigger.

HEV was the most frequent infectious trigger, followed by EBV, *Mycoplasma pneumoniae*, SARS‐CoV‐2, parvovirus B19 and VZV (Figure 1). In the NA group, 8/57 cases (14%) had a positive HEV IgM serology (Table 2), compared with none in the matched controls. Serum HEV RNA was detected by RT‐PCR in four cases.

Laboratory signs of acute hepatitis (increased levels of serum ALT and/or GGT) were found in 13 patients, of whom eight had a preceding infection (five HEV, one EBV, two *Mycoplasma pneumoniae*; Table 3). None of the NA cases and of the controls had two (or more) concomitant recent infections.

**TABLE 3:** Patients with laboratory proven hepatitis (values of ALT above 50 U/L and/or GGT above 71 U/L).

Patient no.	Immunological trigger	ALT (U/L)	GGT (U/L)
21	No infection	384	534
49		33	111
54		48	82
33		148	158
13		76	82
8	HEV	120	192
14		58	102
55		897	172
18		394	148
34		639	93
24	EBV	281	184
45	*Mycoplasma pneumoniae*	91	471
57		132	59

Abbreviations: ALT, alanine aminotransferase; EBV, Epstein–Barr virus; GGT, gamma‐glutamyltransferase; HEV, hepatitis E virus.

Eleven patients reported clinical symptoms suggestive of an acute infection in the month preceding NA onset (five patients had fever, four angina, one diarrhoea, one cough), but our laboratory diagnostic panel could not identify the responsible pathogens.

### Clinical features

Acute viral infection was laboratory proven in 9/14 (57.1%) NA cases with bilateral involvement, compared to 6/43 (11.6%) cases with unilateral involvement (*p* = 0.003, Cramèr's *V* = 0.43, moderate to large association) (Table 4). Six of eight (62.5%) HEV IgM positive NA cases had bilateral involvement of the brachial plexus (*p* = 0.002, Cramèr's *V* = 0.47). The OR for developing NA symptoms after a viral exposure was 14 (*p* = 0.0008). ORs could not be computed for other exposure types.

**TABLE 4:** Laterality of NA symptoms and infections, *n* (%).

NA type	Viral infections		Bacterial infections		HEV	
	No	Yes	No	Yes	No	Yes
Unilateral	37 (64.9%)	6 (10.5%)	43 (75.4%)	–	41 (71.9%)	2 (3.5%)
Bilateral	5 (8.8%)	9 (15.8%)	12 (21%)	2 (3.6%)	8 (14%)	6 (10.6%)

*Note*: Fisher's exact test for viral infections, *p* = 0.003 (Cramèr's *V* = 0.43, moderate to large association); for bacterial infections, *p* = 0.057 (Cramèr's *V* = 0.33, small to moderate association); for HEV alone, *p* = 0.002 (Cramèr's *V* = 0.47, moderate to large association).

Abbreviations: HEV, hepatitis E virus; NA, neuralgic amyotrophy.

## DISCUSSION

In 1948, Parsonage and Turner described NA onset occurring in army soldiers during the convalescent stage of several infections [8]. In the worldwide largest cohort, based on patient reports, van Alfen et al. [1] identified as antecedent events a possible infection in 43% and a vaccination in 4.3% of NA cases, but laboratory findings could prove the infection only in about 5% of the cases. An immune trigger was identified in 38.6% of the NA study population. This percentage is higher compared to that previously reported in the Netherlands [1], probably due to our prospective design and the systematic laboratory screening for infectious agents.

HEV infection was the most frequent trigger (14%), expanding previous observations on the association between acute HEV and NA [9, 10, 11].

Data on other pathogens are scant and mostly derived from case reports on Lyme disease [12, 13], acute EBV infection [14, 15, 16], parvovirus B19 [17, 18, 19, 20], VZV [21, 22, 23, 24], CMV [25, 26, 27], *Mycoplasma pneumoniae* [9, 10, 11] and *Bartonella henselae* [28, 29]. No acute *Bartonella* or HIV infection was detected in our cohort, but in the literature several bilateral NA cases have been described concomitantly with *Bartonella* infections [28] or HIV seroconversion [30, 31].

Since our study was conducted during the COVID pandemic, it was expected that a potential role of SARS‐CoV‐2 as a potential trigger of NA would be observed. A systematic review of two databases (LitCOVID and the World Health Organization database on COVID‐19) found 26 adult cases of NA preceded by SARS‐CoV‐2 infection [32]. Another systematic literature review described 36 NA cases related to COVID‐19 infection. Eight of these had bilateral symptoms (22.2%), and most cases had a good recovery [33]. In our cohort the only case with concomitant COVID‐19 infection (SARS‐CoV‐2 RT‐PCR positive) was a 28‐year‐old female with onset of unilateral symptoms after 2 days of high fever and cough. Of note, her mother was diagnosed with NA 2 days after receiving the second dose of COVID‐19 vaccine (mRNA‐1273). The possibility of an underlying genetic predisposition to NA, with greater susceptibility to self‐immune damage, is an observation that will require future investigations.

In our NA cohort, COVID‐19 vaccination represented a far more frequent trigger (12.3%) than COVID‐19 infection (1.7%; Table 2). This proportion is higher compared to that observed in the Dutch cohort [1], where only 4.3% of the patients received a vaccination. This difference is probably due to the rigorous COVID‐19 vaccination campaign in Switzerland between 2021 and 2022. A more recent publication based on pharmacovigilance data described the reporting frequency of NA after COVID‐19 vaccines to be similar to that of other viral vaccines (0.01%) [34].

In the present study, none of the NA cases received any vaccinations other than the COVID‐19 vaccines. As already reported by Ameer et al. [33], our NA cases post COVID‐19 vaccination showed also a mild clinical course.

The pathogenic mechanism by which immune agents trigger NA is still unknown. So far, attempts to detect specific antibodies were unsuccessful [1]. Data from our and previous cohorts [35] show a wide variability of potential preceding infectious agents, but with a narrow time window, suggesting an immune‐mediated, para‐infectious pathogenesis of NA. Laboratory signs of acute hepatitis (increased levels of serum ALT and/or GGT) were found in eight patients with a preceding infection (Table 3), an observation that deserves more attention.

In a large multicentre retrospective study it was shown that NA associated with a preceding HEV infection has more commonly a bilateral brachial plexus involvement, with potential damage of the phrenic nerve [36]. With the current study, this knowledge is expanded observing that also other viruses (EBV, parvovirus B19) and *Mycoplasma pneumoniae* can cause a bilateral NA, in addition to a laboratory proven acute hepatitis. Notably, all these agents are intracellular and therefore may potentially start similar pathways in the immune system.

The strengths of our study are its prospective design with matched controls, the collection of biological samples in the acute phase and before treatment onset, and the standardization of microbiological tests. The main limitations are the limited sample size and the fixed panel of pathogens tested that did not allow the putative infectious agent to be identified in 11/57 (19.3%) cases. This panel of infectious agents was selected based on reported frequency in the literature, feasibility and reliability of the serological test in the available biological material (i.e., serum), and possible clinical consequences for the patient (e.g., the start of an antiviral therapy). Nevertheless, there is no doubt that NA can be potentially triggered by other agents not tested in this study.

In conclusion, this prospective study identified an immune trigger in 22/57 (38.6%) cases. Acute HEV infection and COVID‐19 vaccination were the most frequent immune triggers. An NA diagnosis may thus hide a systemic infection caused by viral or bacterial intracellular agents, especially in cases of bilateral involvement of the brachial plexus and/or laboratory confirmed hepatitis. It is believed that increasing the awareness of immune triggers preceding NA can speed up the diagnostic process, motivating other colleagues (e.g., hepatologists, internal medicine specialists etc.) to refer patients in the acute phase to the neurologist for diagnostic confirmation. Vice versa, neurologists visiting a new patient with NA should be aware that potentially there is an underlying treatable (e.g., with antiviral agents) systemic infection.

Finally, a better knowledge of the link between infections and autoimmunity could help identify new treatment strategies for NA, but further investigations with larger cohorts are needed to confirm and expand our observations.

## AUTHOR CONTRIBUTIONS

**Davide Sparasci:** Conceptualization; writing – original draft; formal analysis; data curation. **Lenka Schilg‐Hafer:** Investigation; writing – review and editing; data curation. **Bettina Schreiner:** Investigation; writing – review and editing. **Olivier Scheidegger:** Investigation; writing – review and editing. **Anne‐Kathrin Peyer:** Investigation; writing – review and editing. **Agustina Maria Lascano:** Investigation; writing – review and editing. **Alex Vicino:** Investigation; writing – review and editing. **Bernhard Friedrich Décard:** Investigation; writing – review and editing. **Pinelopi Tsouni:** Investigation; writing – review and editing. **Andrea Monika Humm:** Investigation; writing – review and editing. **Enea Pianezzi:** Investigation; writing – review and editing. **Giulia Zezza:** Investigation; writing – review and editing. **Thomas Hundsberger:** Investigation; writing – review and editing. **Anelia Dietmann:** Investigation; writing – review and editing. **Hans H. Jung:** Investigation; writing – review and editing. **Thierry Kuntzer:** Investigation; writing – review and editing. **Einar Wilder‐Smith:** Investigation; writing – review and editing. **Gladys Martinetti‐Lucchini:** Investigation; writing – review and editing. **Orlando Petrini:** Methodology; formal analysis; supervision; data curation; writing – review and editing. **Stefano Fontana:** Investigation; writing – review and editing. **Peter Gowland:** Investigation; writing – review and editing. **Christoph Niederhauser:** Investigation; writing – review and editing. **Claudio Gobbi:** Investigation; writing – review and editing; supervision. **Paolo Ripellino:** Writing – original draft; conceptualization; investigation; writing – review and editing; project administration; data curation; supervision.

## FUNDING INFORMATION

Neuromuscular Research Association, Basel; Foundation for the Advancement of Neurology, Lausanne; Baasch‐Medicus Stiftung, Zurich.

## CONFLICT OF INTEREST STATEMENT

Authors do not report anything to disclose.

## ETHICS STATEMENT

The study was approved by all concerned local Ethics Committees (study no. CE 2932). Written informed consent was obtained from all study participants. Blood donors signed the General Consent of the Blood Transfusion Services of the Swiss Red Cross.

## Data Availability

Anonymized data will be shared on request with any qualified investigator.
